# 676. Assessment of Sequential Testing for *C. Difficile* infections Utilizing Nucleic Amplification Test (NAAT) and Confirmatory Cytotoxic Assay on Diagnostic Stewardship Interventions

**DOI:** 10.1093/ofid/ofad500.738

**Published:** 2023-11-27

**Authors:** Jennifer L Johnson, Ann Vizina, Laura Hempel, Ryan Kuhn, Sorabh Dhar

**Affiliations:** John D. Dingell, VAMC, Brownstown, MI; John D Dingell VAMC, Detroit, Michigan; John D Dingell VAMC, Detroit, Michigan; John D Dingell VA Medical Center, Detroit, Michigan; Wayne State University/Detroit Medical Center, John Dingell VAMC, Detroit, Michigan

## Abstract

**Background:**

*C. difficile* infection (CDI) is a common health care-associated infection and quality measure for hospitals. The use of diagnostic stewardship interventions have been shown to decrease the rates of CDI by guiding appropriate testing using nucleic acid amplification tests (NAAT), which are highly sensitive but cannot differentiate between colonization and infection (i.e. lack clinical specificity). Cell culture cytotoxic assay (the “gold standard” test) has limited use in real time diagnosis, however may play a role in validating and augment the impact of diagnostic stewardship reductions in CDI.

**Methods:**

This single center, retrospective review evaluated the utilization of a cytotoxic assay on clinical patient stool samples that met the criteria for diagnostic testing using NAAT as part of a diagnostic stewardship intervention for appropriate *C. difficile* testing. Samples that were positive for NAAT from 9/11/2018 to 4/11/2023 were concurrently sent for cell culture cytotoxic assay to verify the clinical suspicion of infection from *C. difficile* and the results reviewed by the multidisciplinary care and quality team for clinical correlation.

**Results:**

Of the thirty-seven specimens that tested positive for CDI by NAAT, twenty-eight tested positive on the cytotoxic assay (a total 75.7% of specimen with positive NAAT had concordance with the cytotoxic assay). After clinical adjudication, a decrease of 13 days of therapy (DOT) for vancomycin, 3 DOT for fidaxomicin, and total cost saving of $739.50 was noted after adjustments to therapy. Average turnaround time for the cytotoxic assay was 7.1 days (SD = 3.8).

**Conclusion:**

While limited as a real-time diagnostic assay for CDI, use of the cell culture cytotoxic assay validates the impact of diagnostic stewardship interventions and improves the specificity of testing when utilized in a sequential testing algorithm with NAAT.

**Disclosures:**

**All Authors**: No reported disclosures

